# Does Surgical Intervention Help with Neurological Recovery in a Lumbar Spinal Gun Shot Wound? A Case Report and Literature Review

**DOI:** 10.7759/cureus.4978

**Published:** 2019-06-23

**Authors:** Kyle W Scott, Denslow A Trumbull, William Clifton, Gazanfar Rahmathulla

**Affiliations:** 1 Neurosurgery, University of Florida College of Medicine, Gainesville, USA; 2 Surgery, University of Florida College of Medicine, Gainesville, USA; 3 Neurosurgery, Mayo Clinic, Jacksonville, USA

**Keywords:** bullet, gunshot wound, decompression, penetrating spine injury, lumbar spine, conus medullaris syndrome, cauda equina

## Abstract

The third leading cause of spinal injuries are gunshot wounds to the spine, accounting for 15.2% of all spinal cord injuries. Treatment for gunshot wound spinal cord injuries (GSWSCI) remains variable, with indications for surgery being controversial. There is no clear evidence or guidelines that can help spine surgeons decide and direct surgical intervention. With the paucity of available literature, we report an interesting case of a gunshot injury to the lumbar spine at L1-L2, discuss the presentation and outcome, and evaluate relevant literature. A 27-year-old incarcerated male patient presented with a conus cauda equina asymmetrical injury involving the lower extremities and required initial medical stabilization in the intensive care unit (ICU). He subsequently underwent delayed surgical treatment with decompression and fragment resection at L1-L2. The patient improved neurologically to the American Spinal Injury Association (ASIA) Classification D and eventually regained nearly all lower extremity neurological function. Despite considerable evidence favoring the conservative management of GSWSCI and the absence of guidelines or recommendations on surgical interventions, our case report demonstrates that surgical intervention in appropriately selected patients can yield good recovery of neurological function and improvement in the quality of life. The key remains careful patient selection, the appropriate location of the retained fragment, and the extent of neurological injury that occurred. We feel surgical decompression and fragment removal, along with debridement, can result in good neurological recovery and long-term outcomes.

## Introduction

Interpersonal violence is an increasingly frequent cause of injury globally, with firearms being responsible for a large proportion of these injuries. According to the National Spinal Cord Injury Statistical Center [1], penetrating spinal cord injuries (SCI) account for 16% of all spinal cord injuries. Gunshot wounds (GSW) are the third leading cause of spinal injuries with 15.2% (accounting for 95% of all penetrating SCI). GSW can injure any part of the body, but spinal cord injuries, termed gunshot wound spinal cord injuries (GSWSCI), can result in significant mortality and morbidity [2]. The extent of the injury is determined by many factors, including the presence of spine contusion, vascular injury, as well as the distance, size, and trajectory of the bullet [3]. There is ongoing uncertainty on how to treat GSWSCI due to the range of injury patterns, historically poor outcomes, and associated significant surgical and postoperative morbidity among these patients [4-5].

## Case presentation

**Figure 1:**
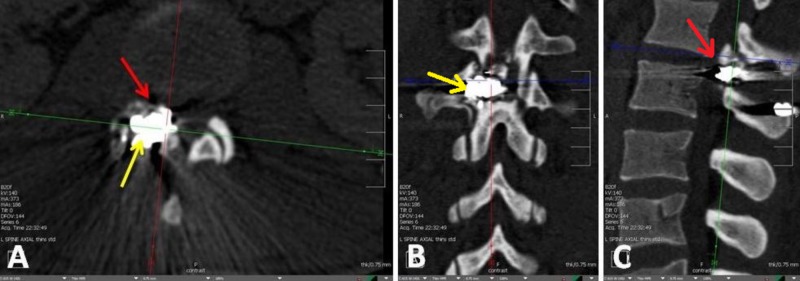
A) Axial CT image of the lumbar spine with bullet fragment lodged on the right side between the L1 and L2 laminae, causing a fracture through the right L2 pedicle with mass effect, compression of the distal roots and conus. In this image, one can view both the large bullet fragment (yellow arrow) and the neuroforaminal and recess compromise (red arrow). B) Coronal plane view. C) Sagittal plane view.

A 27-year-old incarcerated male patient was brought to the emergency department (ED) with a GSW to the lumbar spine. He was systemically unstable, requiring admission to the intensive care unit (ICU) for methicillin-resistant Staphylococcus aureus (MRSA) bacteremia and sepsis. Neurologically, he was conscious and alert, with a Glasgow Coma Score (GCS) of 15 and had urinary and bowel incontinence at presentation, paresthesias, and bilateral leg weakness, indicating a conus medullaris cauda equina syndrome (CMS-CES) injury at the L1-L2 level [6]. While lying supine, the patient demonstrated complete right leg paralysis, with the ability to move his toes and left leg strength of 3/5, but he was only being able to lift his leg approximately 1 inch and not being able to hold the position for more than a few seconds. He had patchy asymmetrical sensory loss with saddle anesthesia. Computed tomography (CT) imaging demonstrated evidence of retained bullet fragments at the level of L1-L2, with fractures present along the right facets and both the right transverse process and the spinous process of L2. These led to severe spinal canal narrowing at the level of L1-L2 and the mass effect and stenosis caused by the bullet fragments resulted in the asymmetric involvement of his lower extremities and his incontinence (Figures 1A-1C).

Once the patient was stabilized, the neurological deficits appeared secondary to mass effect, compression, and stenosis at the level. We decided to decompress and resect the fragment due to the posterior accessible location, corresponding weakness in the right lower extremity, and the incontinence.

**Figure 2:**
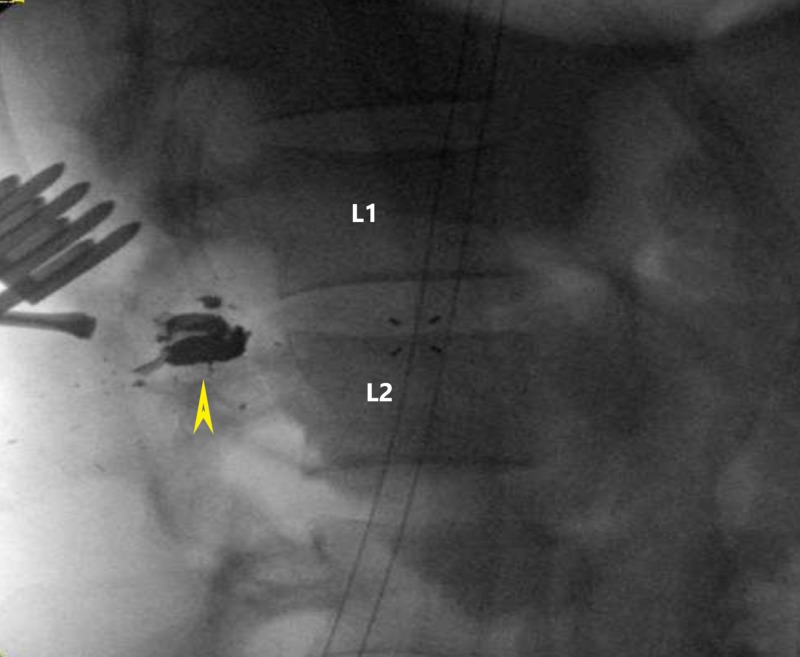
Intraoperative X-ray revealing the location of the bullet fragments (yellow arrowhead) and their anatomical relation to L1-L2, canal, and neuroforamen.

**Figure 3:**
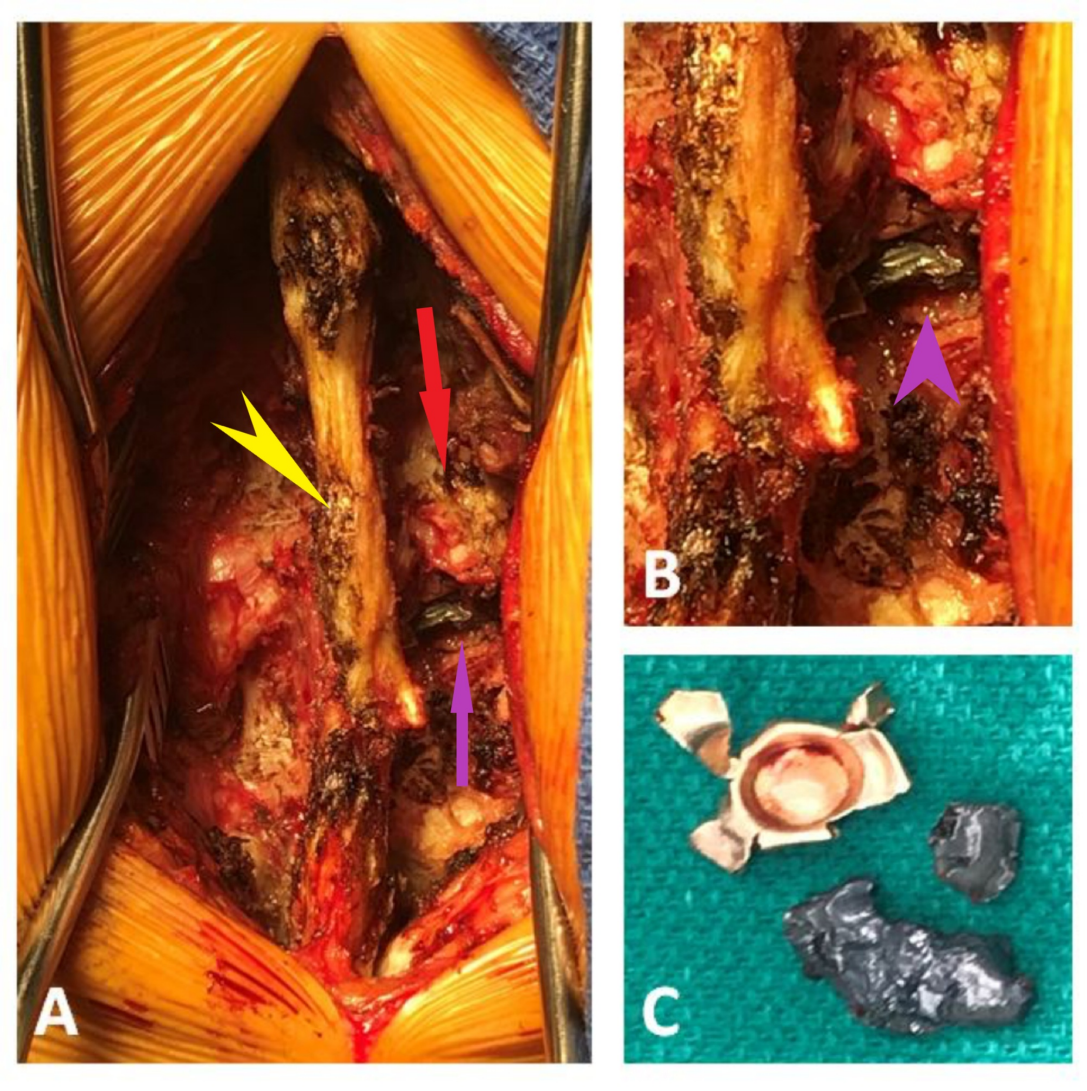
A) Exposed spinous process (yellow arrowhead) and lamina facet (red arrow) revealing the embedded bullet fragment (purple arrow). B) Magnified view of the right L1-2 region clearly revealing the embedded fragment (purple arrowhead). C) Bullet metal fragment and the metallic bullet cap lodged in the lamina.

A standard posterior midline approach was used to access the L1-L2 region. The T12, L1, and L2 spinous processes were excised and a T12 to L2 laminectomy was performed (Figure 2). The bullet fragments were identified in the paraspinal muscle, with a bullet cap and fragments lodged in the facet lamina junction and penetrating the spinal canal, causing compression (Figures 3A-3C). The bullet fragments and fractured bone fragments were excised, and wide decompression was performed without an obvious cerebrospinal fluid (CSF) leak and evident compression and scarring down of the nerves and dural tube in this region. The neural elements were gently freed of all debris and fragments.

In the immediate post-operative period, the patient demonstrated neurological improvement in motor function, with the ability to bear weight and ambulate on the right lower extremity at discharge and an improvement in bowel and bladder function. About two weeks following surgery, the patient exhibited complete recovery of the left lower extremity and near complete recovery of strength in the right lower extremity. At six weeks, he had near complete resolution of symptoms with minimal residual weakness in the lower extremities, improving from CMS-CES to having a substantial gain of autonomic function and the ability to void urine and bowel control. He did have autonomic sexual dysfunction during his recovery.

## Discussion

In our particular case, we decided to proceed with an open surgical approach and perform bullet fragment debridement and spinal decompression as we felt the mass effect combined with the spinal stenosis were responsible for his neurological deficits and could yield better neurological outcomes. The accessible location, focal neurological deficit greater on the side where the fragment was lodged, and the incomplete nature of the patient's neurological injury made us strongly consider surgical intervention. In certain cases of GSWSCI, spinal instability caused by a blast injury from a bullet fragment may require consideration for surgical intervention and stabilization, especially in cases where the morphology of the GSWSCI characterizes it as being unstable. The main factors unique to this patient included the location of the bullet fragment, the presence of incomplete versus complete neurological injury, and the purported mechanism of neurological injury (mass effect of the fragment versus a blast injury to the neural structures or bone as the cause of the impairment).

The standard treatment has been controversial among spine surgeons due to an absence of strong evidence. In 1991, Waters et al. [7] published a reliable study concerning GSWSCI. Outside of a multitude of case reports, comprehensive reviews of GSW treatment standards have been missing [4-5]. Recently, there has been an increase in such studies contributing to the ongoing debate relating to GSWSCI management. The different modalities of treatment include a conservative approach and a surgical approach aimed at minimizing the neurological deficit. The presence of a CSF leak from the bullet wound site or active infection from bullet fragments is commonly used as a metric for performing surgical bullet fragment removal and debridement [8]. A conservative approach can range from observation to spine stabilization using thoraco-lumbo-sacral orthosis [8-9]. Some authors contend that it is the various additional vectors, forces, and trajectories at initial injury, such as the fragment lodged site, bullet trajectory, blast effect, and initial neurological presentation, with complete or incomplete spinal cord injury, along with criteria for spinal instability, which determine the outcome rather than the surgical intervention [3,10]. However, further evaluation of existing literature reveals inconclusive evidence that surgery leads to more complications and is not associated with an improvement in neurological outcomes [5,11-13]. Contradicting arguments remain with surgery being recommended in certain clinical situations, including the presence of spinal instability. Rare situations exist where an incomplete spinal cord injury coexists with the compressing effect of a bullet fragment, and, in these rare instances, surgical decompression of the neural components, with procedures attempting to remove fragments, can likely improve neurological outcomes [7,13-17]. Controversies abound on factors such as the timing of surgery, type of surgery, requirements for spinal stabilization, antibiotics, steroids, and acute versus delayed management of penetrating GSWSCI, with limited literature addressing these issues and requiring further investigation [17]. Steroids were not used in the management of this patient, as there is no strong evidence showing their benefit in these cases [18]. There is a paucity of literature regarding the dose, type, and route of steroid in cases of GSWSCI. Our patient received a five-day course of broad-spectrum antibiotics along with treatment for his MRSA infection on admission. The precise usage of antibiotics in these patients is debated and varies based on the trajectory of the bullet. Studies have shown that in the absence of a trans-gastrointestinal trajectory, there may be no benefit from antibiotic usage [19]. A trans-gastrointestinal trajectory with likely perforation of a hollow viscus would have signaled a longer course of antibiotics covering gut flora due to increased rates of infection [19]. Additionally, the initial management of the trans-gastrointestinal trajectory would have required medical stabilization and sepsis evaluation as necessary steps and necessitates an exploratory laparotomy, repair of intra-abdominal visceral or vascular structures based on the findings, and, if possible, bullet debridement. Although this is a single case reported from our institution, it supports a growing body of literature recommending surgical decompression as a viable option in carefully selected cases of incomplete neurological injuries with radiological evidence of significant spinal cord compromise from mass effect and compression along with a surgically accessible bullet fragment location [4,16]. These considerations have to be taken into account in the overall management of these challenging injuries.

Our case demonstrates the possibility of neurological recovery, which is likely to be variable and patient factor dependent. The appropriate and careful selection of cases remains the most important factor, considering the risks associated with surgical intervention may be significant in comparison to the benefits of surgery. Prognostication of the degree of neurological recovery to the patient and family necessitates caution. Surgery may not benefit GSWSCI with diffuse blast injuries, fragments that have traversed the spinal canal or cord, and patients with complete neurological deficits. The nature of spinal gunshot injuries makes randomized control trials a non-viable option, but institutional collaborative prospective and retrospective data collection and studies will guide treating physician teams with evidence-based management to treat this devastating pathology.

## Conclusions

This case report demonstrates that carefully selected cases of gunshot wounds to the spine may benefit from surgical intervention, with a likelihood of improvement in neurological function and quality of life. The surgical decompression of neural elements, debridement, repair, and stabilization of the spine may assist in optimal neurological recovery in these devastating spinal injuries.
